# A Contribution to the Ætiology of Diphtheria

**Published:** 1901-05-11

**Authors:** W. Hind


### Hospital Clinics and Medical Progress.

### A CONTRIBUTION TO THE ÆTIOLOGY OF DIPHTHERIA.

By W. Hind, M.D., B.S., F.R.C.S., F.G.S.

In May, 1897, a residential school for the education of the blind and deaf was started at The Mount, Stoke-on-Trent, under a combined authority appointed by the majority of the School Board of North Staffordshire. The site consisted of an old residential mansion which was turned into offices and the master's house, and an extensive series of new buildings erected over portions of the garden and yards of the old house. The subsoil was a reddish, very porous sandstone of upper coal-measure age, and the buildings were situated about 550 feet above sea level and well exposed to wind on all sides and some distance from any other dwellings. The deaf and blind schools are quite distinct, and, of course, the girls' and boys' playrooms and dormitories, but all the children meet in a central hall for meals.

The children, teachers, and staff assembled in May, 1897, and there was little or no sickness until February, 1898. About three weeks after return from the Christmas holidays a deaf boy was sent into the sick ward with a sore throat and high temperature; this case was followed on February 17th by two others, and from this time a continual crop of sore throats occurred one after the other till May 24th, all the cases being in deaf lads. There were twenty-seven cases in all, one lad being attacked twice and one three times. The treatment adopted was isolation, antiseptic swabbing, &c.

I reported the fact of an epidemic to the conjoint board, pointing out that although the school consisted of deaf boys, deaf girls, blind boys, blind girls, the outbreak was confined to deaf boys. Some slight sanitary defects were found and remedied, and the ground was opened, but no cases whatever occurred between March 24th and April 18th. But when the ground was filled in again and drains covered cases again began [to occur. On May 6th, 7th, 8th three cases, one of them occurring in the lad who had furnished the first case, showed signs of definite membrane, and they were removed at once to the district infectious hospital. A consultation was held with the medical officers of health to the Bow County Council, myself, and the architect. The drains were thoroughly tested and found to leak in places. On recommendation the drains were thoroughly overhauled, and a great portion relaid, the children being sent home for holidays for that purpose. All the cases with membrane (four) sent away to the infectious diseases hospital made very speedy recoveries, were not treated with antitoxine, and had no paralyses or secondary symptoms whatever.

The school reassembled again in August, 1898, and on the 11th a deaf girl complained of sore throat and was isolated; on August 15th three deaf boys also complained and were isolated; on the 16th a deaf girl, and on the 18th a blind boy were attacked; the latter showing membrane was removed to the infectious diseases hospital. Between August 22nd and 24th eleven cases of sore throat occurred, one only being a blind boy. The others were all deaf boys. One of these cases was attacked for the third time, and this lad had been sent once to the infectious hospital as having a membrane in his throat. One case was attacked for the fourth time. On August 25th hree deaf girls and one deaf lad were attacked.

On August 26th a blind lad was attacked, and from this time secretion from all (I think I am correct in saying all) throats was examined at the bacteriological department of Mason's College, under the very useful and enlightened arrangement made by the Staffordshire County Council This case was found to contain the bacillus of diphtheria. Four other cases—three in deaf boys, one a return case in a deaf girl—occurred between August 29th and August 31st; one, a fourth attack, including supposed membranous diphtheria. Diphtheria bacillus was found only in this and one other throat.

I had suggested to the school authority that some cesspool or drain belonging to the old mansion had not been found and had been built over, and was a probable cause of the continuance of the trouble after the whole sanitary system had been rearranged and relaid, but the architect assured us that all old drains had been carefully followed and removed, but no cesspit or outfall had been found. It was due to the energetic master of the school that, searching for a cause, he thought he smelt some bad air at one corner of the school near the boys' playroom. He dug down at this spot and found an old drain which had been broken into for a foundation and left. This was followed under the deaf boys' playground for several yards. Immediately the ground was opened to remove this old drain, sore throats vanished; but the work being finished, the ground was filled in and reasphalted. No sooner was this the case than sore throats began again, and five cases occurred between September 11th and September 21st, four being in deaf boys, two cases being fifth attacks, and one in a deaf girl. The ground was now opened again to search for a cesspit or old disused drains, and remained open some time; but the work being done, the trenches were filled in and the playgrounds reasphalted. At once sore throats began to recur, and between October 25th and November 2nd, seven cases were isolated, two deaf boys, three blind boys, and one deaf girl. The diphtheria bacillus was found in the case of the deaf girl only. The absence of sore throat during interferences with drains and whenever the ground was open for exploration was so marked that I reported on the fact to the school authority, asking them to provide for some permanent method of ventilating the ground below the asphalt and concrete layers on which the school was built. This was done, and all trouble stopped at once for a time—*i.e.* between November 2nd, 1898, and March 29th, 1899, no cases of sore throat were found to occur.

The noticeable feature about this epidemic of sore throat was first the very large percentage of deaf boys affected; this was accounted for by the fact that the old sealed drains were found under their playground and under their workshop. The cases were, for some time, limited to deaf boys, but it is not clear how the blind boys became attacked, as they were placed in quite another part of the house. The deaf girls probably contracted the complaint from the methods employed in oral instruction. No blind girls were attacked. Another interesting fact is the number of cases who had return attacks. Two boys had five attacks each, each being sent away at least once for diphtheria to the infectious diseases hospital. The comparatively slight nature of the disease—only lasting, as a rule, three to five days—and the rapid recovery in all cases without sequelæ, paralysis, glandular enlargement, or kidney affection, and the fact that attacks did not render the children immune are to be noted. As far as I remember antitoxin was used in no case whatever. The typical bacillus was found in very few cases. Clinically, the cases were follicular tonsillitis, and all those showing membrane were considered to be diphtheria.

It seemed possible to me that the explanation of this epidemic was due to the fact that old cultivated and contaminated soil was sealed by a layer of cement and asphalt; that as long as there was no settling in the new building and it was air-proof below all was well; for the place had been used for nine months before an outbreak. Cold weather came on, and in January, 1898, some settlement had occurred, disturbing drains and allowing slight fissures to form. Fermentation went on in the ground owing to absence of ventilation, and the effete products escaped into the part of the building occupied by the deaf boys. Possibly there was some throat to throat infection, and possibly, in the earlier cases, owing to the rapid recovery, they were not isolated long enough or antiseptic swabbing persevered with sufficiently.

After an interval of several months three cases of sore throat occurred between March 29th and April 11th, 1899—one deaf girl, one blind boy, and one blind girl. No diphtheria bacillus was found in either case. In September a blind girl complained; in November two deaf lads were isolated; but no other cases occurred during the whole year.

After the Christmas holidays the school continued healthy till March, when I saw a male pupil teacher who had a high temperature and sore throat. He taught blind children only, but both sexes.

Bacteriological examination showed the presence of diphtheria bacilli, and he was removed to the infectious hospital. On March 12th one blind girl, and on March 15th a blind boy and a deaf girl were sent to the sick bay with sore throats and a high temperature, and diphtheria bacilli were cultivated from the secretions from their throats. They were all sent to the infectious hospital, and made a rapid and speedy recovery, not followed by sequelæ.

It seemed to me that a new element had arisen, as the attack had singled out a set of children who had been previously unaffected. I therefore held a throat parade, and selected all children who had any chronic enlargement of tonsils, or submaxillary glands, or injected throats, whose temperatures were found to be normal, and who made no complaint and took swabs from all these throats for culture. At the same time I took swabs from the throats of eight members of the teaching staff, and from that of the matron, all of which were sent to Mason's College bacterological laboratory without any indication as to clinical symptoms. The results were very remarkable. About 50 per cent. of the apparently healthy children's throats gave cultivations, and about 70 per cent. of the teachers connected with the deaf as well as the blind also gave evidence of the presence of the diphtheritic bacillus. One was the singing mistress. In one case, a teacher of the blind, the throat was stated to be highly virulent, the cultivation giving excessive results, and this was in a young lady who had a few months previously gone through an attack of acute pneumonia. All teachers' throats, and all children's throats which had given cultivations were swabbed for a fortnight with antiseptics, and no more cases occurred. Two cases of catarrhal sore throat which happened in June and gave no bacteriological results should be mentioned. Writing now, March 1901, I am glad to say that no sore throats have occurred in the school for nine months.

No doubt this epidemic was due to infection from throat to throat, probably the singing mistress being the original culprit. The lady happened to be one of the healthiest people in the place, and was probably immune to the bacillus, which on the other hand could not have been of a very virulent type. It is remarkable and worthy of note to what an extent the deaf escaped. These children of course do not learn singing, but the methods of oral teaching necessitates interference with the lips and tongue and close contact with the exhaled air of a teacher's breath. Little wooden spatulas are used by the teachers to model the lips and tongue and to place these vocal organs in the proper position to form the various sounds. Hence a great danger of infection in teaching the deaf. The master has at my suggestion set up glass jars of disinfectants in each class room in which the spatula is placed after use with each child, and in this way I hope to obviate any danger.

The two epidemics, evidently different in origin, are highly instructive each in its own way, and point out how easily a widespread epidemic of diphtheria may arise from unsuspected causes. I believe a troublesome epidemic in another large school was traced to the singing mistress who herself did not suffer. If these brief notes prove of service in drawing attention to possible causes of infection during times of epidemic sore throat, they will have fulfilled the writer's purpose.

